# Evidence of Microbial Translocation Associated with Perturbations in T Cell and Antigen-Presenting Cell Homeostasis in Hookworm Infections

**DOI:** 10.1371/journal.pntd.0001830

**Published:** 2012-10-04

**Authors:** Palakkal Jovvian George, Rajamanickam Anuradha, Nathella Pavan Kumar, Vasanthapuram Kumaraswami, Thomas B. Nutman, Subash Babu

**Affiliations:** 1 National Institutes of Health, International Center for Excellence in Research, Chennai, India; 2 National Institute for Research in Tuberculosis, Chennai, India; 3 Laboratory of Parasitic Diseases, National Institute of Allergy and Infectious Diseases, National Institutes of Health, Bethesda, Maryland, United States of America; 4 SAIC-Frederick, Inc., NCI-Frederick, Frederick, Maryland, United States of America; University of Edinburgh, United Kingdom

## Abstract

**Background:**

Microbial translocation (MT) is the process by which microbes or microbial products translocate from the intestine to the systemic circulation. MT is a common cause of systemic immune activation in HIV infection and is associated with reduced frequencies of CD4^+^ T cells; no data exist, however, on the role of MT in intestinal helminth infections.

**Methods:**

We measured the plasma levels of MT markers, acute-phase proteins, and pro- and anti - inflammatory cytokines in individuals with or without hookworm infections. We also estimated the absolute counts of CD4^+^ and CD8^+^ T cells as well as the frequencies of memory T cell and dendritic cell subsets. Finally, we also measured the levels of all of these parameters in a subset of individuals following treatment of hookworm infection.

**Results:**

Our data suggest that hookworm infection is characterized by increased levels of markers associated with MT but not acute-phase proteins nor pro-inflammatory cytokines. Hookworm infections were also associated with increased levels of the anti – inflammatory cytokine – IL-10, which was positively correlated with levels of lipopolysaccharide (LPS). In addition, MT was associated with decreased numbers of CD8^+^ T cells and diminished frequencies of particular dendritic cell subsets. Antihelmintic treatment of hookworm infection resulted in reversal of some of the hematologic and microbiologic alterations.

**Conclusions:**

Our data provide compelling evidence for MT in a human intestinal helminth infection and its association with perturbations in the T cell and antigen-presenting cell compartments of the immune system. Our data also reveal that at least one dominant counter-regulatory mechanism i.e. increased IL-10 production might potentially protect against systemic immune activation in hookworm infections.

## Author Summary

Hookworm infections affect more than half a billion people worldwide and cause morbidity in the form of intestinal injury and blood loss. Host immunologic factors that influence the pathogenesis of disease in these individuals are not completely understood. Circulating microbial products such as LPS and markers associated with microbial translocation (transfer of microbes or microbial products from the intestine to the circulation) have been shown to play an important role in disease pathogenesis of certain infections like HIV. We have attempted to elucidate the role of the above mentioned factors in disease pathogenesis by comparing the plasma levels of the various markers in a group of hookworm infected and uninfected individuals. We show that circulating levels of microbial translocation markers are elevated in hookworm infected individuals, a potential cause of morbidity in these infections. This is associated with changes in the host immune system, especially in terms of lymphocyte and dendritic cells subsets. However, microbial translocation is not accompanied by increased levels of acute phase proteins or pro-inflammatory cytokines indicating that the parasite has evolved mechanisms to dampen LPS induced inflammation. Thus, our study highlights a novel pathway of pathogenesis in an intestinal helminth infection and improves our understanding of the various factors involved in the complex host-parasite interaction.

## Introduction

Microbial translocation (MT) is the process by which microbes or microbial products—such as lipopolysaccharide (LPS) and bacterial DNA—translocate from the intestinal lumen to the systemic circulation in the absence of overt bacteremia [1]. Activation of Toll-like receptors by LPS is then thought to lead to systemic immune activation [1]. LPS and 16 s ribosomal RNA (common to most bacteria) are often used as indicators of MT, while soluble CD14 (sCD14) and LPS-binding protein (LBP) are used to establish evidence of direct LPS stimulation [1], [2]. Presence of anti-LPS core antibodies (Endo core LPS antibody, or EndoCAb) is also used as a surrogate measure of circulating LPS [1], [2]. MT is commonly observed in conditions associated with disruption of the gastrointestinal (GI) epithelial barrier such as inflammatory bowel disease, graft-versus-host disease, and chronic viral infections including human-immunodeficiency virus (HIV) and hepatitis C virus [1], [2]. Although MT is known to occur in infections affecting the integrity of the gut epithelium [3], [4], very few studies have examined the occurrence of this phenomenon in intestinal helminth infections.

Hookworm infections are common intestinal helminth infections (affecting 740 million people worldwide) known to cause intestinal injury and blood loss [5]. Hookworm infection in humans is caused by the helminth parasites *Necator americanus* and *Ancylostoma duodenale*. Infection is acquired by entry of infective-stage larvae through the skin during contact with contaminated soil, followed by larval migration through the heart and lungs and subsequent development into adults in the GI tract. The adults then produce eggs, which are deposited in the feces and develop into infective larvae, completing the life cycle. The host must therefore mount an immune response against a number of different life-cycle stages during a hookworm infection [5], [6]. In addition, due the chronic nature of this infection, the parasite has been postulated to manipulate the host immune system to establish long-standing infection [6].

MT is also commonly associated with acute and chronic systemic immune activation and perturbations in T cell subset numbers [1], [2]. Thus, in HIV infection, circulating LPS is associated with increased secretion of proinflammatory cytokines and decreased frequencies of CD4^+^ T cells as well as the selective loss of Th17 (CD4^+^IL-17^+^) T cells [7], [8]. To explore the relationship of MT, innate and adaptive immune homeostasis, and immune activation with hookworm infection, we measured markers of MT and acute-phase proteins, pro- and anti- inflammatory cytokines along with CD4^+^, CD8^+^ T cell, NK cell, and B cell numbers as well as frequencies of CD4^+^ and CD8^+^ T cell and dendritic cell (DC) subsets in hookworm-infected (INF) and uninfected (UN) individuals. Our study provides evidence for the occurrence of MT in hookworm infection associated with perturbations in immune cell compartments. Our study also reveals that MT in hookworm infections (unlike HIV) does not directly translate to systemic immune activation, perhaps due to counter-regulatory measures, such as increased IL-10 production, induced by the parasite.

## Materials and Methods

### Study population

We prospectively studied a group of 46 INF and 45 UN individuals in an area endemic for hookworm infections in Tamil Nadu, South India (table 1). This was a community-based study, and all individuals were recruited from the same village in Tamil Nadu and were of similar socio-economic status. Blood (total volume of 10 ml with or without anti-coagulants) and stool samples were collected from all recruited individuals within the same time period. All INF individuals were treated with a single dose of albendazole (400 mg). Follow-up blood and stool samples were obtained from 30 of the treated individuals 3 months following treatment. All individuals were examined as part of a clinical protocol approved by Institutional Review Boards of both the National Institute of Allergy and Infectious Diseases and the National Institute for Research in Tuberculosis (NCT00375583), and informed written consent was obtained from all participants.

**Table 1:** Demographics and hematology profile.

	UNINF (n = 45)	INF (n = 46)	p value
Hgb gm/DL (Range)	15.18 (10–18.4)	12.9 6.8–17.6)	p<0.0001
RBC 10^6^/µL GM (Range)	5.02 (3.62–5.93)	4.57 (2.59–6.28)	p = 0.0016
WBC 10^3^/µL GM (Range)	7.84 (4.3–15.6)	9.05 (4.7–19.1)	p = 0.0086
HCT % (GM Range)	41.09 (27.8–50.5)	37.19 (22.3–50.2)	p = 0.0045
PLT 10^3^/µL GM (Range)	282.68 (189–555)	294.96 (168–781)	NS
Neutrophil 10^3^/µL GM (Range)	4.35 (2.35–10.35)	4.51 (1.95–14.7)	NS
Lymphocyte 10^3^/µL GM (Range)	2.51 (1.56–4.7)	2.45 (1.06–3.93)	NS
Monocyte 10^3^//µL GM (Range)	0.52 (0.26–1.23)	0.53 (0.24–1.53)	NS
Eosinophil 10^3^/µL GM (Range)	0.25 (0.08–0.81)	1.05 (0.19–4.64)	p<0.0001
Basophil 10^3^/µL GM (Range)	0.06 (0.02–0.29)	0.06 (0.02–0.21)	NS

RBC, red blood cell; WBC, white blood cell; HCT, hematocrit; NS, not significant.

### Parasitologic examination

Single stool samples were collected, transported to the laboratory at ambient temperatures, and examined by direct microscopy and by formal-gasoline concentration techniques, as described previously [9]. Stool microscopy was used to exclude the presence of other intestinal helminths including *Ascaris, Strongyloides, Trichuris, Enterobius, Taenia* and *Hymenolepis*. Concomitant filarial infection was excluded by the TropBio Og4C3 enzyme-linked immunosorbent assay (ELISA) (Trop Bio Pty. Ltd, Townsville, Queensland, Australia).

### Hematologic analysis

Hematology was performed on all patients using the Act-5 Diff hematology analyzer (Beckman Coulter, Brea, CA, USA).

### MT markers

To inactivate plasma proteins, plasma samples were heated to 75°C for 5 min. LPS levels were measured using a limulus amebocyte lysate assay (Cell Sciences Hycult Biotech, Canton, MA, USA) according to the manufacturer's protocol. Commercially available enzyme-linked immunosorbent assay (ELISA) kits were used to measure plasma levels of lipid-binding protein (LBP), endotoxin core antibodies IgG (EndoCAb), intestinal fatty acid binding protein (IFABP), (all Cell Sciences Hycult Biotech), and sCD14 (R&D Systems, Minneapolis, MN, USA).

### Acute-phase proteins

Plasma levels of C-reactive protein (CRP), haptoglobin, serum amyloid A (SAA), and α-2 macroglobulin (α-2M) were measured using the Bioplex multiplex ELISA system (Bio-Rad, Hercules, CA, USA) according to the manufacturer's instructions.

### Cytokines

Plasma levels of cytokines, TNF-α, IFN-γ, IL-12, IL-17 and IL-10 (Bio-Rad) were measured using the Bioplex multiplex ELISA system in a subset of samples.

### Flow cytometry analysis

Flow cytometry acquisition was done on BD FACS Canto II (BD Biosciences, San José, CA, USA). Analysis was done using FlowJo software v9.4.10 (TreeStar Inc., Ashland, OR, USA). T, B and NK cells were enumerated in whole blood using BD Multiset 6-Color TBNK cocktail (BD Biosciences). Naïve and memory T cell phenotyping (Figure S1) as well as dendritic cell subset phenotyping (Figure S2) were performed using lineage specific antibodies (BD Pharmingen and eBioscience).

### Statistical analysis

Data analyses were performed using GraphPad PRISM (GraphPad Software, Inc., San Diego, CA, USA). All samples were tested in duplicate by ELISA. Geometric means (GM) were used for measurements of central tendency. Statistically significant differences were analyzed using the nonparametric Mann-Whitney U test and Wilcoxon matched pair test. Multiple comparisons were corrected using the Holm's correction for each set of analysis. Correlations were calculated by the Spearman rank correlation test.

## Results

### Hookworm infection is associated with an increased frequency of eosinophils but decreased frequencies of neutrophils, lymphocytes, and monocytes

As shown in table 1, INF individuals differed from UN individuals in exhibiting significantly lower hemoglobin levels (*P*<0.0001), hematocrit (*P* = 0.0045) and red blood cell counts (*P* = 0.0016). In contrast, INF individuals had significantly increased numbers of eosinophils (*P*<0.0001) and total white blood cell counts (*P* = 0.0086).

### Hookworm infection is associated with elevated levels of LPS, sCD14, EndoCAb, and IFABP

**Figure 1:**
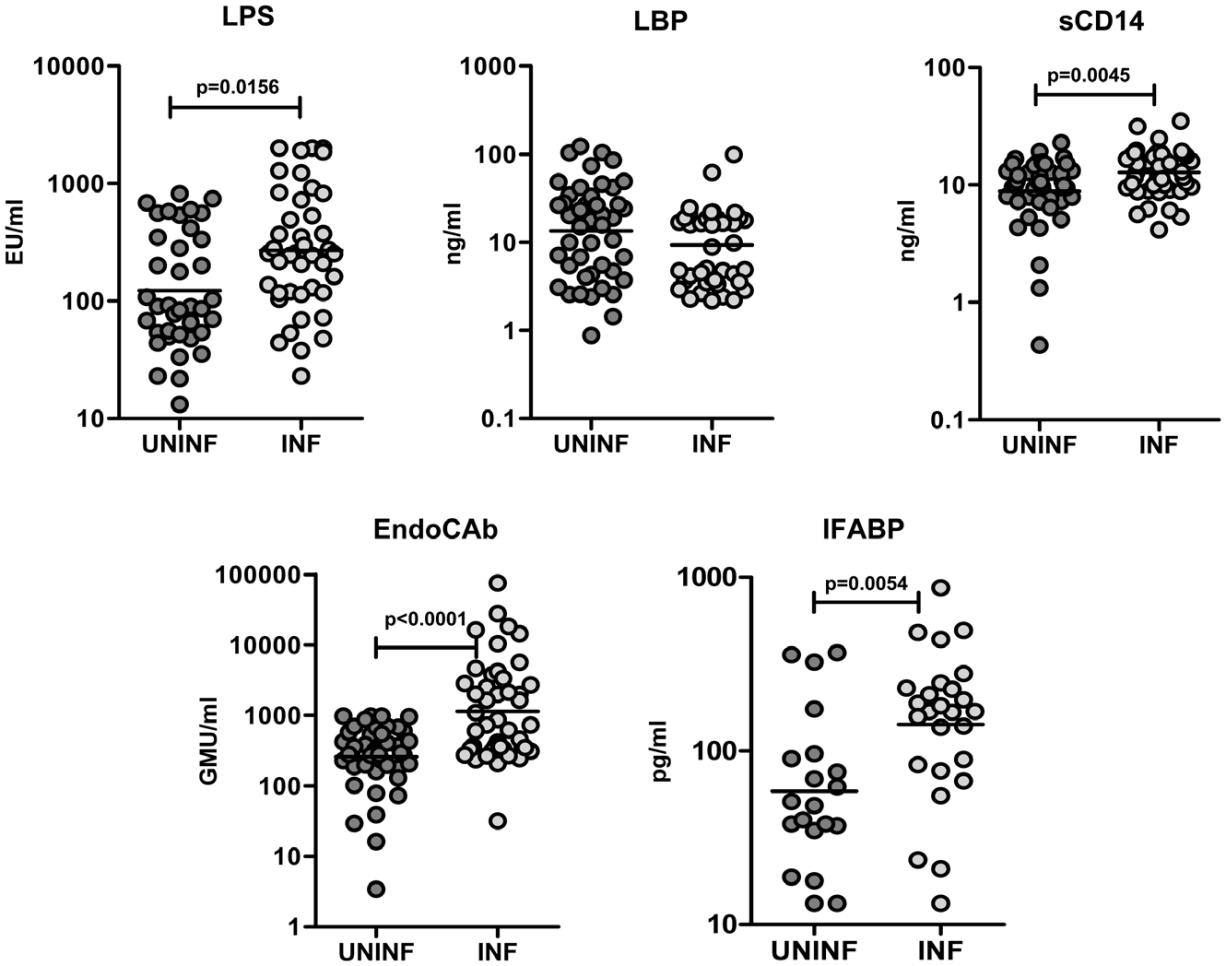
Hookworm infection is associated with elevated levels of LPS, sCD14, EndoCAb, and IFABP. Plasma levels of lipopolysaccharide (LPS), lipid binding protein (LBP), soluble CD14 (sCD14), endotoxin core antibody IgG (EndoCAb), and intestinal fatty acid binding protein(FABP) from hookworm-infected [INF] (n = 27–46) or uninfected [UNINF] (n = 20–45) individuals were measured by ELISA. *P* values were calculated using the Mann-Whitney test.

To determine the association of MT and related markers with hookworm infection, we measured the plasma levels of LPS, LPB, sCD14, EndoCAb, and IFABP in INF and UN individuals. As shown in figure 1, INF had significantly higher levels of LPS (GM of 271 EU/ml in INF vs. 122.8 in UN; *P* = 0.0156), sCD14 (GM of 12.8 ng/ml in INF vs. 8.8 in UN; *P* = 0.0045), EndoCAb (GM of 1132 GMU/ml in INF vs. 257.5 in UN; *P*<0.0001), and IFABP (GM of 141.8 pg/ml in INF vs. 58.5 in UN; *P* = 0.0054) in comparison to UN. Thus, hookworm infection is associated with elevated circulating levels of molecules often associated with MT.

### Hookworm infection is associated with decreased levels of CRP, haptoglobin, IL-17 and increased levels of IL-10

**Figure 2:**
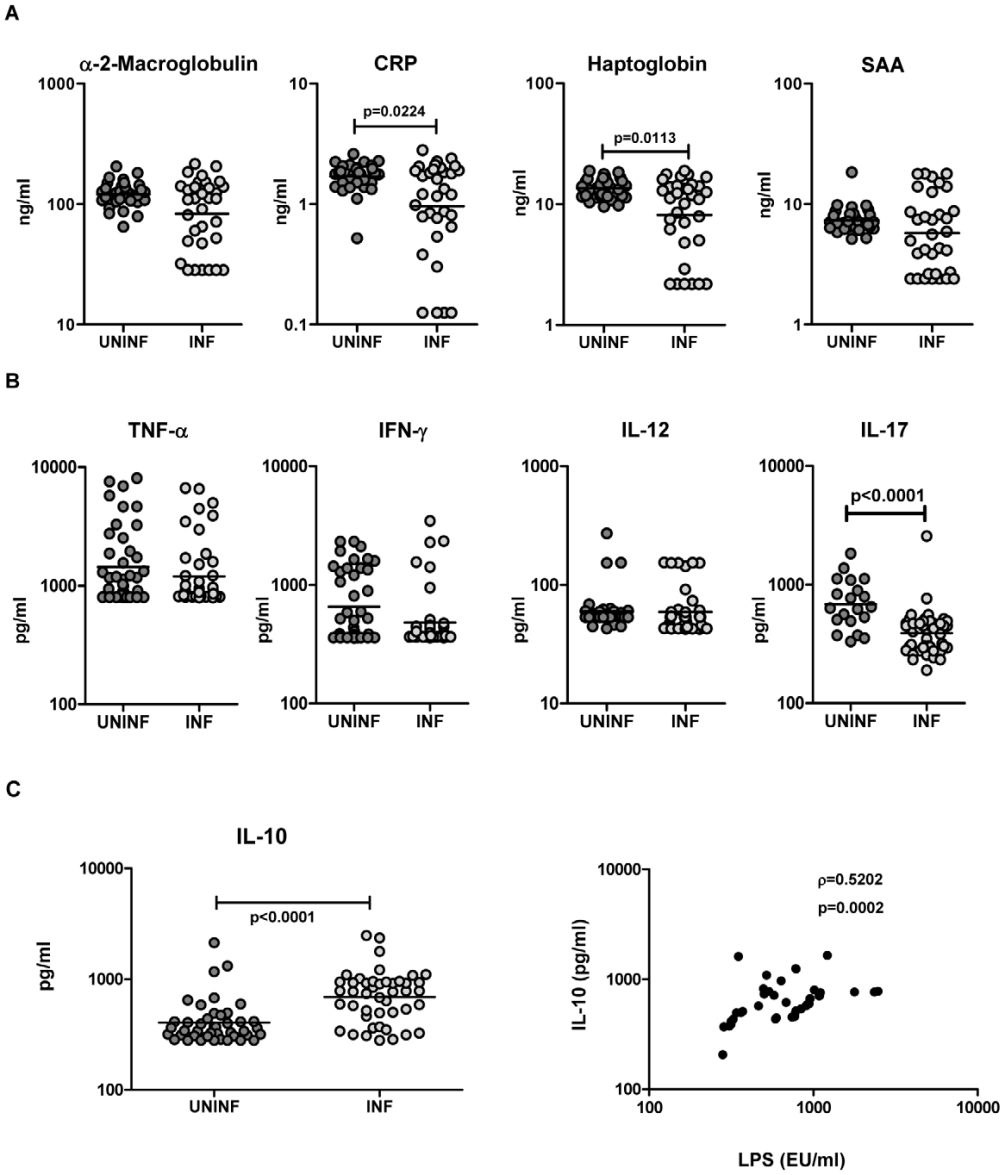
Hookworm infection is not associated with elevated levels of acute-phase proteins or pro-inflammatory cytokines. *A*, Plasma levels of α-2 macroglobulin, C-reactive protein (CRP), haptoglobin, and serum amyloid proteins (SAA) from hookworm-infected [INF] (n = 33) or uninfected [UNINF] (n = 33) individuals were measured by ELISA. *B*, Plasma levels of TNF-α, IFN-γ, IL-12, and IL-17 from INF (n = 45) or UNINF (n = 20–42) individuals were measured by ELISA. (C) Plasma levels of IL-10 from INF (n = 45) and UNINF (n = 42) individuals were measured by ELISA. Plasma levels of LPS were correlated with levels of IL-10 in INF (n = 45) individuals. *P* values were calculated using the Mann-Whitney test and Spearman rank correlation test.

To determine the association of acute-phase proteins with hookworm infection, we measured the plasma levels of α-2M, CRP, haptoglobin, and SAA in INF and UN individuals. As shown in figure 2A, INF had significantly lower levels of CRP (GM of 0.95 ng/ml in INF vs. 1.7 in UN; *P* = 0.0224) and haptoglobin (GM of 8.1 ng/ml in INF vs. 13.5 in UN; *P* = 0.0113) but not α-2M and SAA in comparison to UN. Similarly, to determine the association of pro-inflammatory cytokines with hookworm infection, we measured plasma levels of TNF-α, IFN-γ, IL-12, and IL-17. As shown in figure 2B—with the exception of IL-17, which was significantly decreased in INF (GM of 390 pg/ml in INF vs. 682.8 in UN; *P*<0.0001)—no significant alterations in plasma levels of pro-inflammatory cytokines were observed in hookworm infection. Conversely, INF had significantly higher levels of IL-10 compared to UN (GM of 689 pg/ml in INF vs. 404 in UN; *P*<0.0001[figure 2C]). Moreover, there was a significant correlation between plasma levels of LPS and those of IL-10 (r = 0.520; *P* = 0.0002) indicating that individuals with increased LPS levels also had increased levels of IL-10. Thus, hookworm infection was not associated with either acute-phase protein elevation or elevation of pro-inflammatory cytokines; there was however concomitant increases in IL-10, which could mitigate the systemic immune activation often associated with MT.

### Hookworm infection is associated with decreased CD8+ T cell counts; reduced CD4+ and CD8+ effector memory T cell frequencies and altered frequencies of DC subsets

**Figure 3:**
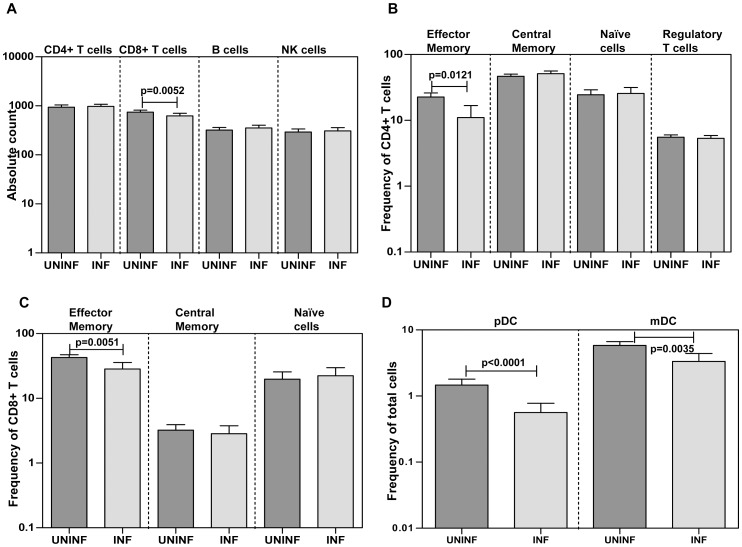
Hookworm infection is associated with decreased CD8^+^ T cells and dendritic cell (DC) subsets. *A*, Absolute counts of CD4^+^ and CD8^+^ T cells, B cells, and NK cells in INF (n = 46) and UNINF (n = 45) individuals were measured by flow cytometry. *B*, Percentages of effector memory (CD45RA^−^CCR7^−^), central memory (CD45RA^−^CCR7^+^), and naïve (CD45RA^+^CCR7^+^) CD4^+^ T cells as well as natural regulatory T cells (CD4^+^CD25^+^Foxp3^+^CD127^dim^) in INF (n = 31) and UNINF (n = 40) were measured by flow cytometry. *C*, Percentages of effector memory (CD45RA^−^CCR7^−^), central memory (CD45RA^−^CCR7^+^), and naïve (CD45RA^+^CCR7^+^) CD8^+^ T cells in INF (n = 31) and UNINF (n = 40) were measured by flow cytometry. (D) Percentages of plasmacytoid (pDC; HLA-DR^+^ CD123^+^) and myeloid DC (mDC; HLA-DR^+^CD11c^+^) in INF (n = 46) and UNINF (n = 45) were measured by flow cytometry. *P* values were calculated using the Mann-Whitney test.

Because MT is associated with decreased CD4^+^ T cell counts in HIV infection [7], we sought to determine the relationship between hookworm infection and/or MT with the numbers of CD4^+^ and CD8^+^ T cells, NK cells, and B cells. As shown in figure 3A, INF had significantly lower numbers of total CD8^+^ T cells (GM of 624 cells/µl in INF vs. 743 in UN; *P* = 0.0052) but not total CD4^+^ T cells, NK cells, or B cells in comparison to UN. To determine the association of MT with alterations in T cell subsets, we measured the frequencies of different CD4^+^ and CD8^+^ T cell subsets in hookworm infection. As shown in figure 3B and C, we observed a significant decrease in percentages of effector memory CD4^+^ (CD45RA^−^CCR7^−^; GM of 28.3% in INF vs. 42.8 in UN; *P* = 0.0121) and CD8^+^ (CD45RA^−^CCR7^−^; GM of 11% in INF vs. 22.5 in UN; *P* = 0.0051) T cells but not central memory (CD45RA^−^CCR7^+^) or naïve T cells (CD45RA^+^CCR7^+^) or nTregs (CD4^+^CD25^+^Foxp3^+^CD127^dim^). We also sought to determine whether hookworm infection is also associated with perturbations in the antigen-presenting cell compartment and hence determined the frequency of the DC subsets. As shown in figure 3D, INF had significantly lower frequency of pDCs (Lin^−^HLA-DR^+^ CD123^+^; GM of 0.56% in INF vs. 1.4 in UN; *P*<0.0001) as well as mDCs (Lin^−^HLA-DR^+^ CD11c^+^; GM of 3.3% in INF vs. 5.8 in UN; *P* = 0.0035) in comparison with UN. Thus, hookworm infection is associated with alterations in homeostatic levels of total CD8^+^ T cells, the relative frequencies of both CD4^+^ and CD8^+^ effector memory T cells as well as baseline frequency of DC subsets.

### Relationships between LPS/EndoCAb and CD8+ T cells and DC subsets in hookworm infection

**Figure 4:**
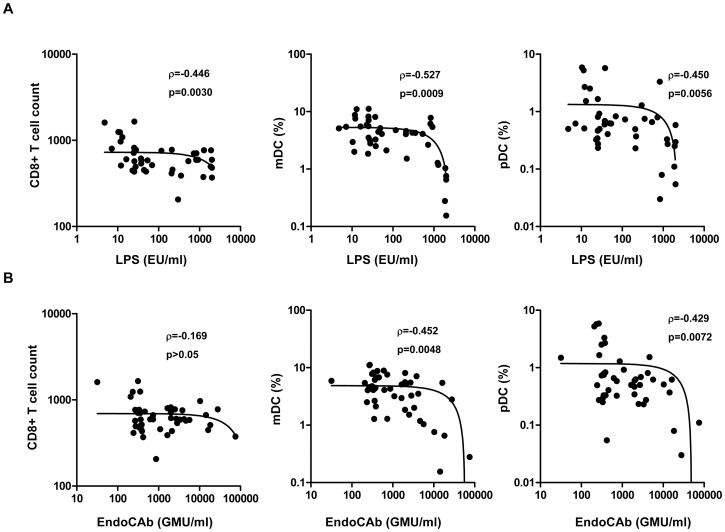
Correlation between microbial translocation markers and CD8^+^ T cells and DC subsets in hookworm-infected individuals. *A*, Plasma levels of lipopolysaccharide (LPS) were correlated with the absolute counts of CD8^+^ T cells and frequencies of myeloid (mDC) and plasmacytoid DC (pDC) in hookworm-infected individuals (n = 46). *B*, Plasma levels of endotoxin core antibody IgG (EndoCAb) were correlated with the absolute counts of CD8^+^ T cells and frequencies of mDC and pDC in hookworm-infected individuals (n = 46). *P* and r values were calculated using the Spearman rank correlation test at 95% confidence intervals.

The relationships between circulating levels of LPS and EndoCAb and CD8^+^ T cells and DC subsets were next assessed in INF individuals (figure 4). As shown in figure 4A, levels of LPS exhibited a significant negative correlation with baseline CD8^+^ T cell counts (r = −0.446; *P* = 0.0030) as well as with mDC (r = −0.527; *P* = 0.0003) and pDC (r = −0.450; *P* = 0.0028) frequency in INF. Similarly, EndoCAb levels were significantly negatively correlated with the baseline frequency of mDC (r = −0.452; *P* = 0.0016) and pDC (r = −0.429; *P* = 0.0036) (figure 4B). No significant correlation was observed between MT markers and T cell subsets in INF individuals. In addition, no significant correlation was observed between these parameters in UN individuals (data not shown). Thus, the process by which MT occurs appears to exhibit a significant negative association with CD8^+^ T cell numbers and mDC and pDC percentages in hookworm infection.

### Treatment-induced alterations in MT markers and DC subset frequency in hookworm infection

**Figure 5:**
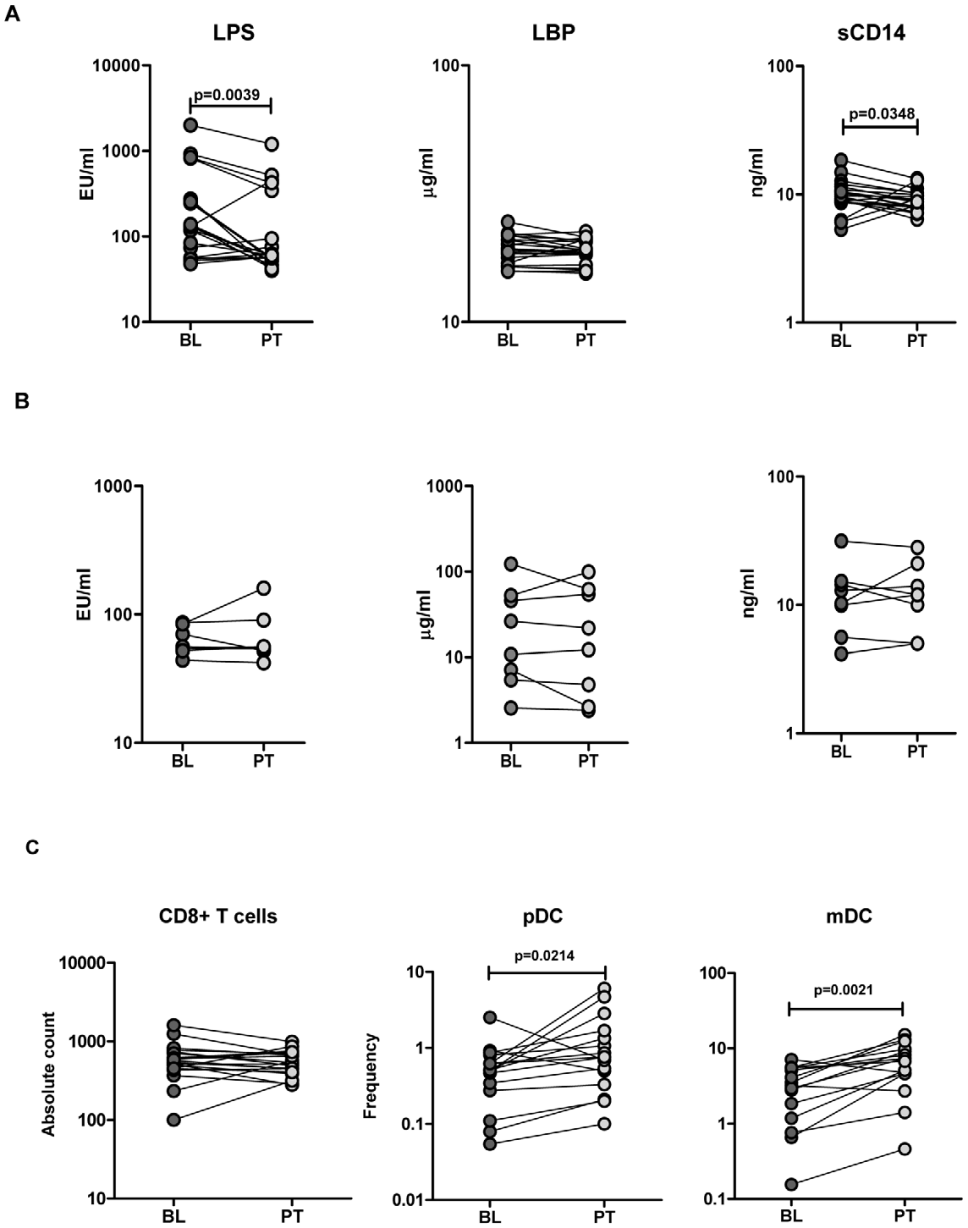
Treatment of hookworm infection is associated with alterations in MTMs and dendritic cell (DC) subsets. *A*, Plasma levels of lipopolysaccharide (LPS), lipid binding protein (LBP), soluble CD14 (sCD14), and endotoxin core antibody IgG (EndoCAb) from hookworm-infected (n = 22) individuals before (BL) and 3 months following treatment (PT) were measured by ELISA. *B*, Plasma levels of LPS, LBP and sCD14 from hookworm-infected (n = 8) individuals, who continued to harbor parasites, before (BL) and 3 months following treatment (PT) were measured by ELISA. *C*, CD8^+^ T cell counts and dendritic cell subset frequencies in hookworm infected (n = 22) individuals before and 3 months following treatment were measured by flow cytometry. *P* values were calculated using the Wilcoxon matched pair test.

Of the 46 INF individuals treated, 30 were able to be assessed at three months following treatment. 22/30 were found to be hookworm free at 3 months with the remaining 8 either still harboring infection or reinfected. As shown in figure 5A, treatment (and resultant cure) of hookworm infections caused a significant decrease in the circulating levels of LPS (GM of 93.6 EU/ml following treatment vs. at 172 baseline; *P* = 0.0039) and sCD14 (GM of 8.8 ng/ml vs. 9.7; *P* = 0.0348) compared to pre-treatment levels. In contrast, in those who failed to cure their hookworm infection there were no significant alterations in the levels of LPS, LBP or sCD14 between those found pre- and 3 months following albendazole therapy (figure 5B). However, it is possible that the lack of statistical power in this group could lead to a lack of change in the markers investigated. Nevertheless, successful treatment of hookworm infection was associated with significant increases in pDCs (GM of 0.79% following treatment vs. 0.42 at baseline; P = 0.0214) and mDCs (GM of 5.4% vs. 2.4; P = 0.0021) at 3 months following anthelmintic therapy (figure 5C).

## Discussion

MT is characterized by translocation of microbial products from the intestinal lumen into the circulation and has been shown to occur as a consequence of disruption to the barrier function of the intestinal epithelium [1]. Studies in experimental animal models suggest that intestinal injury and systemic endotoxemia are two major factors leading to morbidity in helminth infections. Infection with the enteric nematodes *Trichinella spiralis* and *Strongyloides venezuelensis* has been shown to be associated with enhanced leakiness of the intestinal epithelium and translocation of LPS into the circulation in experimental animal models [3], [4]. This leakiness—mediated in part by activated mast cells—can lead to movement of bacterial LPS into the portal circulation [3], [4]. Even in non-intestinal helminth infections, such as *Schistosoma mansoni*, in which adult parasites reside in the mesenteric veins, damage caused by worm eggs traversing the GI epithelium can result in systemic translocation of bacteria [10], [11], [12]; however, no study has examined the role of MT in a human intestinal helminth infection. Because morbidity from hookworm infections is directly related to intestinal injury and blood loss caused by attachment of worms to the intestinal mucosa and submucosa [5], hookworms are likely helminth candidates to induce MT. Therefore, the present study sought to elucidate the systemic effects of MT in hookworm infection.

We examined five important circulating microbial or related products in our study. LPS (a key indicator of MT) was found to be significantly elevated in INF individuals. This was accompanied by a significant increase in levels of sCD14, EndoCAb, and IFABP. Although increased levels of LBP are a common feature of MT in other infections [7], [13], [14], we did not observe any significant alteration in LBP levels in hookworm infections. LPS is commonly measured to assess quantitatively the degree to which MT and plasma LPS levels are directly associated with the degree of intestinal permeability following invasive GI surgery [15]. Similarly, sCD14, a soluble receptor for LPS produced by monocytes and macrophages, is often increased following MT and is felt to be an indicator of LPS stimulation in vivo [16]. Naturally occurring IgG antibodies to the LPS core oligosaccharide are potent neutralizers of LPS activity and are commonly elevated following systemic endotoxemia [17], [18]. Finally, IFABP is an intracellular epithelial protein in the stomach and small and large intestinal mucosa and appears in the circulation after epithelial damage. Hence, plasma IFABP levels are considered useful markers for early diagnosis of intestinal ischemia [19]. Our data suggest that IFABP may also be a potentially reliable marker of the breach in epithelial integrity associated with chronic intestinal infections. We have not performed a quantitative assessment of the parasite burden in the stool of these individuals and some of the differences observed within the infected group could potentially reflect infection intensity, while some of the UN individuals may have had light infections. Nevertheless, our study groups did not differ significantly in age, gender, socio-economic status and were from the same geographical area, indicating that these confounding factors may not play a significant role.

Circulating microbial products are well known inducers of acute-phase proteins, with SAA and haptoglobin known to be markedly elevated following challenge with LPS [20]. Acute-phase proteins derive primarily from the liver, and plasma concentrations are felt to be a reflection of the response to pro-inflammatory cytokines [21]. Because MT is commonly associated with systemic immune activation, we measured plasma levels of acute-phase proteins α-2M, CRP, haptoglobin, and SAA. Our data show that hookworm infections are not associated with significant elevations in acute-phase proteins. Indeed, CRP and haptoglobin levels were actually significantly lower in INF individuals. To confirm that MT in hookworm infections is not associated with systemic inflammatory responses, we also measured plasma levels of pro-inflammatory cytokines. Our examination of cytokine expression levels revealed that pro-inflammatory cytokines are not significantly elevated in INF individuals, again confirming the lack of a systemic inflammatory milieu associated with MT in hookworm infection. This is in agreement with the cytokine profiles observed in previous studies of experimental human infection [22], [23], [24]. Although studies in HIV infection reveal a direct association between levels of MT markers such as LPS and pro-inflammatory cytokines [7], [13], the lack of systemic pro-inflammatory cytokines may be reflective of counterbalancing by regulatory cytokines or Treg populations commonly seen in chronic helminth infection [25], [26]. While increased levels of IL-10 production in hookworm infected individuals has been observed before [23], [27], our data on the increased levels of IL-10 in INF suggest that at least one potential mechanism by which systemic immune activation fails to occur in hookworm infections might be due to the heightened levels of IL-10. Indeed, IL-10 is known modulator of LPS induced systemic immune responses and protects against lethal damage and septic shock [28], [29]. Our data also suggest that natural regulatory T cells may not play a significant role in dampening inflammation in hookworm infections (see Fig. 3B). Again, this is in contrast to a previous report that suggested that regulatory T cells are present at increased frequencies in hookworm infections [30]. In addition, other parasite dervived products, such as the production of proteins, termed “helminth defense molecules,” that have been shown to actively inhibit LPS-induced inflammation [31], might also contribute to the absence of overt systemic inflammation.

MT is associated with perturbed CD4^+^ T cell homeostasis in HIV infection and idiopathic lymphocytopenia [7], [32]. Although the exact mechanism by which CD4^+^ T cell depletion is associated with MT is still unclear, it is widely believed that depletion of the intraepithelial and lamina propria CD4^+^ T cells could disrupt the integrity of the intestinal epithelium [1]. Interestingly, in hookworm infections, we observed significant perturbations in not only the CD8^+^ T cell compartment but also in frequencies of specific DC subsets. Our data on the lack of difference in the circulating levels of CD4^+^ T cells is in contrast to previous studies that had shown lower levels of CD4^+^ T cells in INF individuals [33], [34]. Our data therefore suggest an important association between MT and homeostatic levels of CD8^+^ T cells and DCs, as we demonstrated a significantly negative relationship between the levels of LPS/EndoCAb and the frequencies of CD8^+^ T cells and those of pDC and mDC. Moreover, we also identified the presence of significantly lower frequencies of both CD4^+^ and CD8^+^ effector memory T cells in hookworm infections, although an association between MT with altered T cell memory needs to be demonstrated. Th17 cells have been shown to play an important role in mucosal defense against bacterial and fungal pathogens and maintenance of the integrity of the mucosal barrier [35]. Selective depletion of Th17 cells in HIV-1 disease has been attributed to MT [8]. While we have not examined Th17 cell frequency directly, we report that hookworm infection is associated with significantly reduced levels of the prototypical Th17 cytokine IL-17. Finally, the lower frequencies of mDCs and pDCs, cells known to normally induce proinflammatory cytokines and chemokines [36], [37] following TLR ligation, may also provide an explanation for the failure of the hookworm-induced MT to induce systemic immune activation.

Having demonstrated that hookworm infection is associated with MT that, in turn, affects certain cell populations, we wanted to examine how treatment/cure of hookworm infection might influence these parameters. Our data on individuals treated for hookworm (and proven cured by absence of eggs in the stool) demonstrate a partial reversal of MT events and a partial reconstitution of the DC compartment. Thus, treatment with albendazole and resultant cure led to diminution of the plasma levels of LPS and sCD14 and to an increase in frequency of pDC and mDC. Although we did not observe a significant reversal in CD8^+^ T cell counts and plasma levels of EndoCAb or pro-inflammatory cytokines (data not shown), our treatment data considerably strengthen the association of hookworm infection with increased gut permeability and altered immune cell homeostasis and argue for a causal relationship.

In summary, our study describes a novel relationship between an intestinal helminth infection and the process of MT—a process that might contribute to immune-mediated pathogenesis. It also suggests new targets for modulation of the pathology associated with hookworm infection.

## Supporting Information

Figure S1 **Gating strategy for estimating frequencies of naïve, central and effector memory T cells and nTregs.** A representative flow cytometry plot showing the gating strategy for estimation of naïve, central memory, and effector memory cells from CD4^+^ and CD8^+^ T cells. Naïve cells were classified as CD45RA^+^CCR7^+^, effector memory cells as CD45RA^−^CCR7^−^, and central memory cells as CD45RA^−^CCR7^+^. nTregs were classified as CD4^+^CD25^+^Foxp3^+^CD127^dim^.
(DOC)

Figure S2 **Gating strategy for estimating frequencies of dendritic cell (DC) subsets.** A representative flow cytometry plot showing the gating strategy for estimation of plasmacytoid (pDC) and myeloid (mDC) dendritic cells from lineage-negative cells. pDC were classified as HLA-DR^+^ CD123^+^ and mDC as HLA-DR^+^CD11c^+^.
(DOC)
