# Supplementary material for: Evidence of Microbial Translocation Associated with Perturbations in T Cell and Antigen-Presenting Cell Homeostasis in Hookworm Infections
Source: PLoS Negl Trop Dis. 2012 Oct 4;6(10):e1830. doi: 10.1371/journal.pntd.0001830 (PMC3464301; doi:10.1371/journal.pntd.0001830)
Supplement: Figure S1 — Gating strategy for estimating frequencies of naïve, central and effector memory T cells and nTregs. A representative flow cytometry plot showing the gating strategy for estimation of naïve, central memory, and effector memory cells from CD4+ and CD8+ T cells. Naïve cells were classified as CD45RA+CCR7+, effector memory cells as CD45RA−CCR7−, and central memory cells as CD45RA−CCR7+. nTregs were classified as CD4+CD25+Foxp3+CD127dim. (DOC) [file pntd.0001830.s001.doc]

Evidence of Microbial Translocation Associated with Perturbations in T Cell and Antigen-Presenting Cell Homeostasis in Human Hookworm Infections

P. Jovvian George,^1^ R. Anuradha,^1^ N. Pavan Kumar,^1^ V. Kumaraswami,^3^ Thomas B. Nutman,^4^ and Subash Babu,^1,2^

^1^National Institutes of Health—International Center for Excellence in Research, Chennai, India, ^2^SAIC‑Frederick, Inc., NCI‑Frederick, Frederick, Maryland, United States of America, ^3^National Institute for Research in Tuberculosis, Chennai, India, and ^4^Laboratory of Parasitic Diseases, National Institute of Allergy and Infectious Diseases, National Institutes of Health, Bethesda, Maryland, USA

**Supplementary Materials**

**Figure S1.** Gating strategy for estimating frequencies of CD4^+^ and CD8^+^ naïve, central memory, and effector memory T cells and nTregs.

**Figure S1.** Gating strategy for estimating frequencies of CD4^+^ and CD8^+^ naïve, central memory, and effector memory T cells and nTregs. A representative flow cytometry plot showing the gating strategy for estimation of naïve, central memory, and effector memory cells from CD4^+^ and CD8^+^ T cells. Naïve cells were classified as CD45RA^+^CCR7^+^, effector memory cells as CD45RA^–^CCR7^–^, and central memory cells as CD45RA^–^CCR7^+^. nTregs were classified as CD4^+^CD25^+^Foxp3^+^CD127^dim^.
