# Supplementary material for: Evidence of Microbial Translocation Associated with Perturbations in T Cell and Antigen-Presenting Cell Homeostasis in Hookworm Infections
Source: PLoS Negl Trop Dis. 2012 Oct 4;6(10):e1830. doi: 10.1371/journal.pntd.0001830 (PMC3464301; doi:10.1371/journal.pntd.0001830)
Supplement: Figure S2 — Gating strategy for estimating frequencies of dendritic cell (DC) subsets. A representative flow cytometry plot showing the gating strategy for estimation of plasmacytoid (pDC) and myeloid (mDC) dendritic cells from lineage-negative cells. pDC were classified as HLA-DR+ CD123+ and mDC as HLA-DR+CD11c+. (DOC) [file pntd.0001830.s002.doc]

Evidence of Microbial Translocation Associated with Perturbations in T Cell and Antigen-Presenting Cell Homeostasis in Human Hookworm Infections

P. Jovvian George,^1^ R. Anuradha,^1^ N. Pavan Kumar,^1^ V. Kumaraswami,^3^ Thomas B. Nutman,^4^ and Subash Babu,^1,2^

^1^National Institutes of Health—International Center for Excellence in Research, Chennai, India, ^2^SAIC‑Frederick, Inc., NCI‑Frederick, Frederick, Maryland, United States of America, ^3^National Institute for Research in Tuberculosis, Chennai, India, and ^4^Laboratory of Parasitic Diseases, National Institute of Allergy and Infectious Diseases, National Institutes of Health, Bethesda, Maryland, USA

**Supplementary Materials**

**Figure S2.** Gating strategy for estimating frequencies of dendritic cell subsets.

**Figure S2.** Gating strategy for estimating frequencies of dendritic cell (DC) subsets. A representative flow cytometry plot showing the gating strategy for estimation of plasmacytoid (pDC) and myeloid (mDC) dendritic cells from lineage-negative cells. pDC were classified as HLA‑DR^+^ CD123^+^ and mDC as HLA-DR^+^CD11c^+^.
